# Low-Cost Online Partial Discharge Monitoring System for Power Transformers

**DOI:** 10.3390/s23073405

**Published:** 2023-03-23

**Authors:** Wojciech Sikorski, Artur Wielewski

**Affiliations:** Institute of Electric Power Engineering, Poznan University of Technology, 60-965 Poznan, Poland

**Keywords:** partial discharge (PD), online monitoring, low-cost system, power transformer, transformer diagnostics, acoustic emission (AE), piezoelectric transducer, microcontroller, arduino, teensyduino

## Abstract

The article presents in detail the construction of a low-cost, portable online PD monitoring system based on the acoustic emission (AE) technique. A highly sensitive piezoelectric transducer was used as the PD detector, whose frequency response characteristics were optimized to the frequency of AE waves generated by discharges in oil–paper insulation. The popular and inexpensive Teensy 3.2 development board featuring a 32-bit MK20DX256 microcontroller with the ARM Cortex-M4 core was used to count the AE pulses. The advantage of the system is its small dimensions and weight, easy and quick installation on the transformer tank, storage of measurement data on a memory card, battery power supply, and immediate readiness for operation without the need to configure. This system may contribute to promoting the idea of short-term (several days or weeks) PD monitoring, especially in developing countries where, with the dynamically growing demand for electricity, the need for inexpensive transformer diagnostics systems is also increasing. Another area of application is medium-power transformers (up to 100 MVA), where temporary PD monitoring using complex measurement systems requiring additional infrastructure (e.g., control cabinet, cable ducts for power supply, and data transmission) and qualified staff is economically unjustified.

## 1. Introduction

The results of the international survey on substation transformer failures published by CIGRE showed that, in most cases, they were caused by partial discharges [1,2]. Apart from the relatively rarely committed serious manufacturing errors (e.g., non-degassed or under-dried insulation system, the use of conductive structural elements such as screws or nuts with sharp edges, metal particles left after assembly works, incorrectly designed elements of the insulation system, etc.), partial discharges are initiated in these areas of the insulating system that are highly moist and degraded as a result of aging processes (pyrolysis, hydrolysis, and oxidation reactions) [3,4,5]. Partial discharges can also be generated in the vicinity of deformed windings due to overvoltage or mechanical shock during transformer transportation. For this reason, the phenomenon of partial discharges is not only the cause of failure but can be, and in fact is, more and more commonly treated as a reliable indicator of the condition of the transformer insulation system [6,7].

For PD detection in laboratory conditions, the conventional electrical method IEC 60270 [8] is used, while in the place of installation of the power transformer, the acoustic emission (AE) or ultra-high frequency (UHF) method is preferable due to the resistance to external electromagnetic interferences [9,10]. These unconventional techniques of PD detection have been adapted to both periodic diagnostic tests and tests carried out in the online monitoring mode [11,12,13]. The main advantage of monitoring over periodic diagnostics is the possibility of immediate detection of PD ignition or an increase in their intensity and, thus, allows operating services to prepare quickly and implement procedures minimizing the risk of failure. Based on the data collected by the monitoring system, one can dynamically change the requirements for periodic diagnostic tests (e.g., their acceleration or postponement) and decide if and when it is necessary to plan downtime and perform maintenance. Some online PD monitoring systems usually allow data exchange with a substation SCADA system. This enables the implementation of advanced inference rules based on statistical or machine learning methods, which allow detection of the relationships between the PD activity and such monitored parameters as voltage, load, oil temperature, tap position of the OLTP, or the amount of hydrogen dissolved in the oil [14,15,16].

In addition to the undoubted benefits of using online partial discharge monitoring systems, a serious obstacle to their dissemination is still a small number of specialized manufacturers, which translates into low supply and the high price of the device. In addition, the price is increased by the high costs of research and development and production. For this reason, only the largest transformers of key importance for the power system are currently equipped with online partial discharge monitoring systems. Therefore, in recent years, there has been an intensification of research work related to the minimization of the costs of PD monitoring. Two main areas of activity can be distinguished in the conducted research. The first one is related to the development of simple and low-cost partial discharge sensors. Castro et al. discussed [17] the possibility of using piezoelectric membranes (buzzers) to detect PD in power transformers based on the acoustic emission method. Piezoelectric membranes are readily available and often cheaper than conventional AE sensors, making their use particularly attractive in applications where several sensors are used. Laboratory tests have shown that a low-cost piezoelectric membrane provides acceptable partial discharge detection sensitivity and can be an alternative to expensive AE sensors. Besharatifard et al. [18] investigated the possibility of replacing piezoelectric technology with microfiber composites (MFCs). Compared to piezoelectric membranes, MFC sensors are slightly more expensive but offer higher sensitivity up to 500 kHz and a longer pulse duration, which can help separate partial discharges from on-site noise. For several years, also in the case of electromagnetic PD detectors operating in the radio frequency bands (HF, VHF, and UHF), research has been carried out to simplify their design and reduce production costs [19,20,21,22]. An example of such designs are miniaturized UHF antennas, which can be mass-produced using printed circuit board (PCB) technology [23]. According to the literature on the subject, the most promising designs of UHF PD detectors, which can be easily manufactured in PCB technology, are microstrip patch antennas [24,25,26], bio-inspired antennas [27,28], meander-line antennas [29], logarithmic spiral antennas [30], Vivaldi antennas [31], different types of Archimedean spiral antennas [32,33], meandered planar inverted-F antennas, and fractal antennas such as Hilbert curve fractal antennas [34,35], Peano fractal antennas [36], H-fractal antennas [37], or Minkowski fractal antennas [38]. In addition to UHF antennas, the most commonly used low-cost electromagnetic PD detectors are high-frequency current transformers (HFCT) and transient earth voltage (TEV) sensors. Their advantage is not only simple construction but also high sensitivity [39,40].

The second area of research concerns the construction of partial discharge monitoring systems based on generally available single-chip microcontrollers, simple USB data loggers, or field programmable gate arrays (FPGA), which are an alternative to expensive, multi-channel data acquisition cards. Saeed et al. [41] presented a supervisory system for PD monitoring designed around the Microchip PIC24EP512GU810 microcontroller, which is characterized by a high speed of 70 million instructions per second (MIPS). The device also offers a satisfying analog-to-digital conversion performance of 1.1 million samples per second with up to four simultaneous channels. This ensures that the fast PD pulses are adequately sampled. Chakrabarty et al. [42] have developed an online partial discharge counting system using a microcontroller and FPGA technology. The signal from the PD detector is sampled at a frequency of 20 MHz by the analog-to-digital converter of the PIC 16F877A microcontroller. The signal is then transmitted to the input of a Xilinx ML405 evaluation board equipped with an FPGA chipset type Virtex-4 (XC4VFX20) that has been programmed to detect and count PD pulses in real time. Chang et al. [43] have developed a reconfigurable partial discharge monitoring system based on FPGA technology. The proposed system uses the AMR Cortex M4 microprocessor to control the system and data analysis, a fast AD9226 analog-to-digital converter, and Xilinx XC6SLX16 field programmable gate arrays with SDRAM memory. The signal processing procedure consists of two steps. In the first step, PD pulses are captured by various PD detectors, quickly converted to digital data by AD9226, and then temporarily stored in SDRAM by an FPGA chip. In the second step, the signals are taken from the SDRAM and transmitted to the microprocessor by the FPGA for further data analysis. Yan et al. [44] presented the PD monitoring and locating system for medium-voltage switchgears, which is based on low-cost TEV detectors. In order to automatically locate the PD source and minimize the number of expensive high-speed acquisition cards, the authors proposed a time-sharing access mechanism, which was implemented by multiple high-frequency surface mounting relays integrated into each TEV detector. Mohamed et al. [45] proposed the use of a spectrum analyzer based on the SDR (software-defined radio) technology to build a cheap and portable online PD monitoring system operating in the VHF/UHF frequency range. A system that is capable of automatically collecting PD data consists of only two hardware components, i.e., a PC/laptop and a portable software-defined radio receiver type Realtek RTL2832U that connects to the computer via a USB interface. 

This article presents in detail the construction of a low-cost, portable PD online monitoring system based on the acoustic emission (AE) technique, which has a chance to fill the gap in the area of inexpensive monitoring systems that can be effectively used in the diagnosis of small and medium power transformers. Currently designed systems and commercial systems available on the market are intended—mainly due to the high price and complicated construction—to monitor large power transformers that play a strategic role in the power system. To the best of the authors’ knowledge, this manuscript is the first complete description of a portable online PD monitoring system that can be assembled from commercially available electronics and development boards compatible with the Arduino architecture. As a module for processing and analyzing AE signals, the Teensy 3.2 board was used. Despite its small size and low price (20USD), it ensures high efficiency of detection and counting of AE pulses generated by partial discharges. Laboratory tests have shown that the system is capable of lossless, real-time counting of up to over 83,000 AE pulses. 

The developed device is the world’s first PD monitoring system equipped with a piezoelectric acoustic emission sensor optimized for the detection of partial discharges in oil–paper insulation. The frequency characteristics and resonant frequencies of the sensor coincide with the frequencies of AE pulses generated by the most dangerous and destructive types of discharges for the transformer insulation system, i.e., inter-turn discharges, creeping and surface discharges. As a result, the presented low-cost monitoring system has a very high sensitivity of PD detection. What is also essential is that the system is in line with the strategy of modern monitoring of power transformers formulated in the Cigre TB 630 brochure published in 2015: *Guide On Transformer Intelligent Condition Monitoring (TICM) Systems*, which assumes the replacement of traditional sensors and transducers with Intelligent Electronic Devices (IED), which are equipped with a microprocessor and are capable of processing raw PD pulses and automatically determining the parameters describing them.

The developed system may contribute to the promotion of the idea of short-term (several days or weeks) PD monitoring, especially in developing countries where, with the dynamically growing demand for electricity, the demand for inexpensive transformer diagnostics systems is also growing. Another area of application is medium power transformers (up to 100 MVA), on which temporary PD monitoring using expensive, often requiring additional infrastructure (e.g., control cabinet, cable ducts for power and data transmission), and qualified staff would be economically unjustified.

This paper is organized as follows. The hardware and software layers of the system are discussed in Section 2, while Section 3 presents the results of laboratory and field tests that allowed us to assess the possibility of using the system for short-term PD monitoring in power transformers. General conclusions from the conducted research are included in Section 4.

## 2. Materials and Methods

### 2.1. General Description

In designing the device, the following assumptions were made: low price and availability of all system components, easy assembly, high sensitivity of PD detection, resistance to external electromagnetic and acoustic disturbances, registration of the PD data in real time, collecting PD data and storing it on a portable, inexpensive storage medium, battery or mains operation, small dimensions and weight, and immediate readiness to work. 

**Figure 1:**
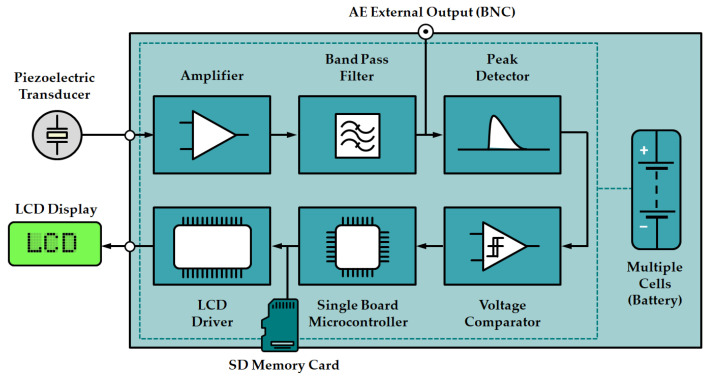
Schematic diagram of a low-cost online partial discharge monitoring system.

The online PD monitoring system is based on the acoustic emission method, which, along with electromagnetic HF/VHF/UHF methods, belongs to the group of unconventional partial discharge detection techniques. The main advantages of the AE method include relatively high sensitivity, which primarily depends on the position of the sensor in relation to the PD source, galvanic separation of the tested object from the measurement system, resistance to external electromagnetic interference, the ability to locate PD using auscultation or TDOA (time difference of arrival) technique, and installation of the measurement system does not require switching off the tested object. As shown in the schematic diagram of the system (Figure 1), a contact piezoelectric transducer was used to detect acoustic waves from PD. The electrical signals at the output of the piezoelectric transducer usually have very small amplitude, from a few to tens of millivolts, therefore, the monitoring system is equipped with a preamplifier circuit consisting of an instrumentation amplifier with adjustable gain (default gain is 40 dB), and a voltage follower. Another element of the system is an active bandpass filter, whose task is to eliminate low-frequency components of the acoustic background (e.g., oil pump and cooling fan noises, vibrations caused by the magnetostrictive action of the transformer core, etc.) and high-frequency electromagnetic interferences. The amplified and filtered signal is then processed by peak detector and voltage comparator circuits. The use of both of these systems made it possible to replace the expensive signal acquisition card with a simple microcontroller equipped with an analog-to-digital converter. The operation of the monitoring system is controlled by a program written in C/C++, which performs such functions as detection and counting PD pulses, saving measurement data on a memory card, and presenting current measurement data on the LCD. 

### 2.2. Hardware Layer

#### 2.2.1. Partial Discharge Detector

**Figure 2:**
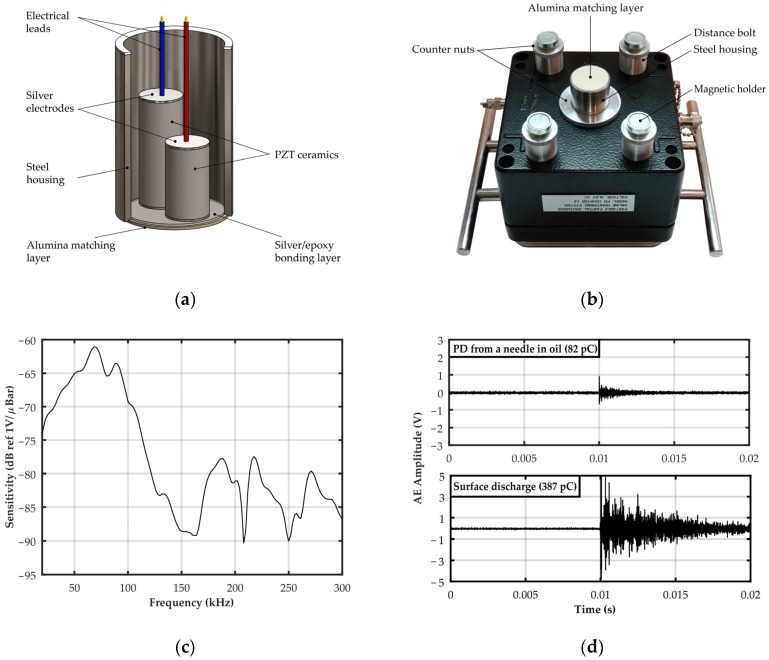
Piezoelectric transducer for detection of AE signals generated by partial discharges: (**a**) schematic diagram of the transducer construction; (**b**) photograph of the monitoring system housing with piezoelectric transducer and magnetic holders; (**c**) frequency response characteristics of the piezoelectric transducer; (**d**) AE waveforms recorded for a PD from a needle in oil with an apparent charge of 82 pC and for a surface discharge on a pressboard sample in oil with an apparent charge of 387 pC.

A contact piezoelectric transducer was used as a partial discharge detector and the various design and production stages, described in detail in Reference [46]. The frequency response of the transducer was determined on the basis of laboratory tests aimed at identifying the acoustic frequencies emitted by partial discharges in oil–paper insulation. The test results showed that the most destructive forms of PD for the transformer insulation system (inter-turn, surface, and creeping discharges) generate AE signals, the energy of which is transferred in three bands: 20–45 kHz, 50–70 kHz, and 85–115 kHz. The dominant frequencies in these bands are 40 kHz, 68 kHz, and 90 kHz, respectively. Since Barkhausen noise from the transformer core can reach ultrasonic frequencies [47], the transducer has been optimized to work in the other two higher frequency bands characteristic for partial discharges, i.e., 50–70 kHz and 85–115 kHz. The optimal material and geometric properties of the main transducer structures were selected using the Krimholtz–Leedom–Matthaei (KLM) model. Based on the simulation results, two piezoelectric disks made of PZT-5A (Navy Type II) ceramics with a diameter of 10.5 mm and a height of 18.8 mm and 25 mm, respectively, were used to build the transducer. Piezoelectric elements with opposite polarization directions were bonded to the matching layer using an electrically conductive composite adhesive based on epoxy resin and silver. The matching layer is made of a round plate with a diameter of 25 mm and a height of 1 mm, made of high-density alumina (Figure 2a). The acoustic impedance of the matching layer is 37.9 MRayl, which ensures an efficient transfer of acoustic wave energy from the mineral oil through the steel tank of the power transformer to the piezoelectric elements of the transducer. Everything is closed in a housing made of stainless steel. The transducer was placed centrally on the front wall of the monitoring system housing, which was additionally equipped with four height-adjustable magnetic holders (Figure 2b).

The selected geometric and material parameters, as well as a fully differential design, allowed to obtain the desired properties of the transducer, i.e., a two-resonant (68 kHz and 90 kHz) and wide (30–100 kHz) frequency response curve and high peak sensitivity (−61.1 dB ref. V/μbar) (Figure 2c). The test results presented in reference [46] showed that this transducer is characterized by high detection sensitivity of partial discharges generated in paper-oil insulation. Compared to commonly used commercial AE sensors, the average PD pulse amplitude recorded by the new transducer was a minimum of 5.2 dB higher and a maximum of 19.8 dB higher. Figure 2d shows the AE waveforms recorded a needle partial discharge in oil with an apparent charge of 82 pC and a surface discharge on a pressboard sample in oil with an apparent charge of 387 pC.

#### 2.2.2. Amplifier and Bandpass Filter

**Figure 3:**
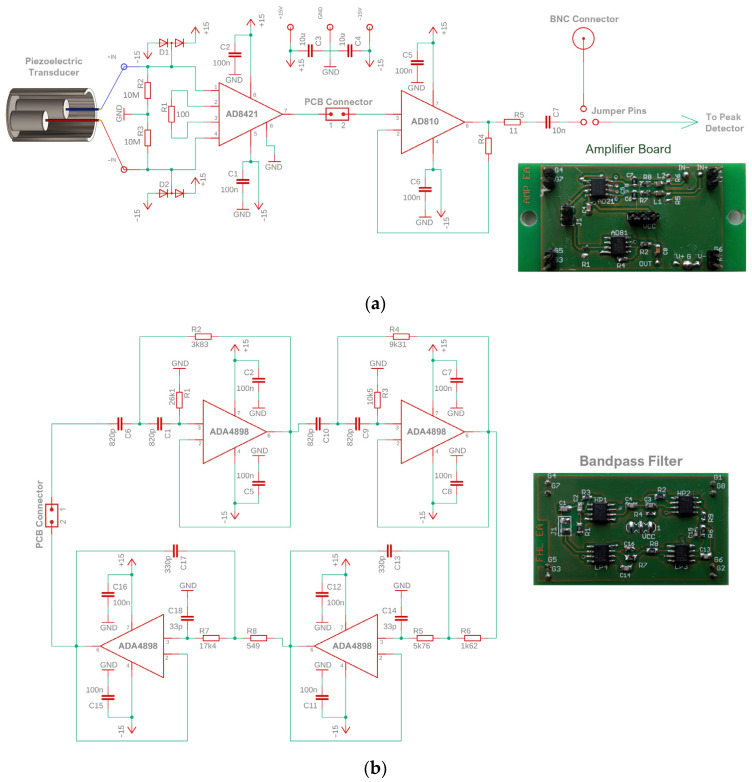
Electronic circuit diagram and photograph of the printed circuit board: (**a**) amplifier, (**b**) bandpass filter.

Figure 3 shows circuit diagrams and photographs of the amplifier and filter designed for the PD monitoring system. The amplifier circuit is based on the AD8421BRZ instrumentation amplifier from Analog Devices (Analog Devices, Norwood, MA, USA). The gain G is regulated by the appropriate selection of the resistance *R_G_* value. In this case, a 100-ohm resistor was used, giving a gain of 40 dB (G = 100). The main advantages of this circuit are low current consumption (<2.3 mA), low noise level, which does not exceed 3.2 nV/√Hz, ultra-low polarization current (<500 pA), wide bandwidth (2 MHz with gain G = 100) and low price (around $10).

The active bandpass filter (20–500 kHz) is designed based on the Sallen–Key architecture, where the low-pass and high-pass sections have a fourth-order Butterworth filter structure with unity gain. For this purpose, the Analog Devices ADA4898 voltage feedback operational amplifier was used, which is characterized by ultralow noise (0.9 nV/√Hz), ultralow distortion (−93 dBc at 500 kHz), and low supply current (8 mA). The filter is made on a separate PCB and is plugged in using dedicated board-to-board connectors between the instrumentation amplifier and the voltage follower. This solution allows the filter to be easily disconnected or replaced with a filter with a different passband characteristic. 

The designed low-cost PD monitoring system provides the possibility of transmitting the amplified and filtered AE signal via a coaxial cable directly to an external measuring device (oscilloscope, signal acquisition card) or to a substation SCADA system. Coaxial cables have a relatively large capacity (50–100 pF/m) and, therefore, cannot be directly connected to the output of the amplifier. This problem was solved by the use of a voltage follower based on the AD810 op-amp, which can drive capacitive loads exceeding 1000 pF, without parasitic oscillations.

#### 2.2.3. Peak Detector and Voltage Comparator

**Figure 4:**
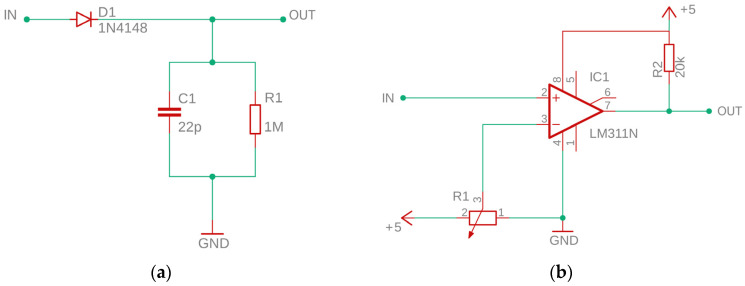
Electronic circuit diagram: (**a**) Peak detector; (**b**) Voltage comparator.

In order to reduce the required sampling frequency of the AE pulses, a passive peak detector (sometimes called an envelope detector) and a voltage comparator were used. The peak detector is made up of just three components: fast-switching diode *D* type 1N4148, capacitor *C* with capacitance 22 pF, and resistor *R* with resistance 1 MΩ (Figure 4a). Together, these elements form a half-wave rectifier that charges the capacitor *C* to the peak voltage of the incoming AE burst. As the amplitude of the input signal increases, the capacitor voltage is increased by a rectifier diode *D*. When the amplitude of the input signal decreases, the capacitor is discharged through a parallel bleeder resistor *R*. The discharge rate of the capacitor depends on the value of the time constant *τ* = *RC*. During the first time constant, the capacitor discharges 63%, and after 5*τ*, it is almost completely discharged.

A voltage comparator is a special type of operational amplifier with an unbalanced input and high gain that compares the voltage value of the signal applied to the non-inverting input with the reference voltage applied to the inverting input. The voltage value at the comparator’s output results from the difference in voltages between its two inputs. If the voltage at the non-inverting input is higher than at the inverting input, then the output voltage is close to the positive pole of the supply. If the voltage at the non-inverting input is lower than at the inverting input, then the output voltage is close to the negative pole of the power supply. Thus, the comparator can be considered an elementary, one-bit analog-to-digital converter. The project uses a single-channel LM311N voltage comparator (Figure 4b), which is characterized by a very low current consumption (up to 100 nA at an ambient temperature of 25 °C) and a fast response time (~200 ns). The open collector (OC) comparator output is compatible with CMOS, TTL, and RTL-DTL integrated circuits and can switch voltages up to 50 V and currents up to 50 mA. The LM311N is designed to operate with a wide range of supply voltages, including ±15 V power supplies for operational amplifiers and, as in this case, 5 V for logic circuits. The voltage value given to the inverting input is set with the A20k rotary potentiometer. 

**Figure 5:**
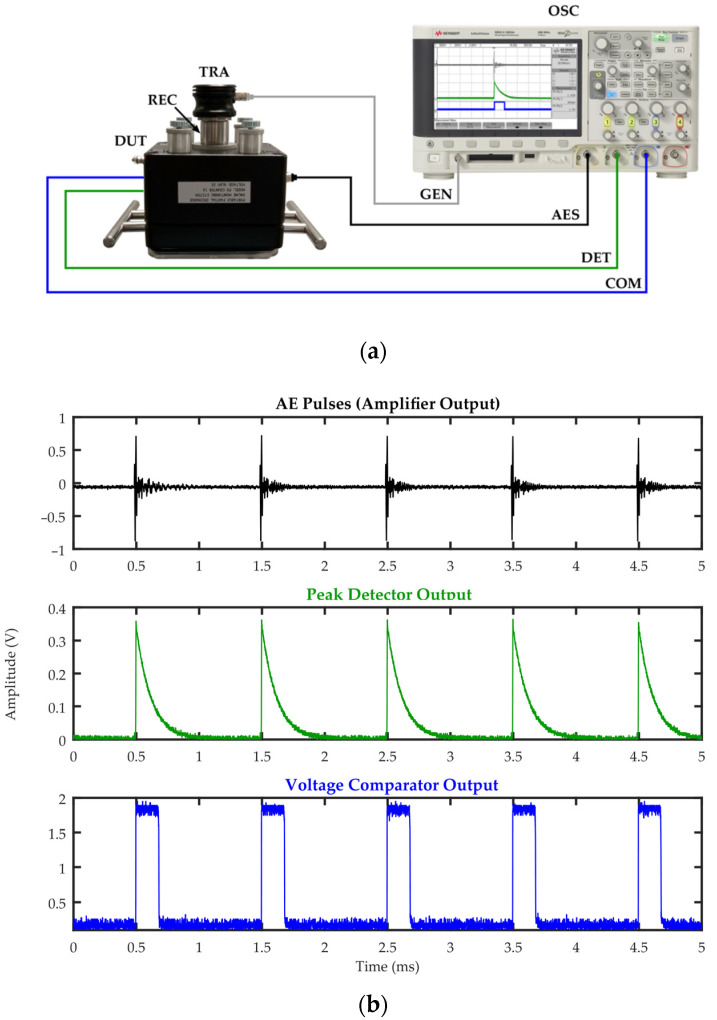
Testing of individual electronic modules of the PD monitoring system; (**a**) schematic diagram of the measurement system: TRA—wideband piezoelectric transducer Olympus V101B for generating AE pulses (transmitter), REC—piezoelectric transducer of the monitoring system (receiver), DUT—system under test, OSC—oscilloscope Keysight DSOX2024A, GEN—signal generator, AES—signal recorded at the output of the amplifier, DET—signal recorded at the output of the peak detector; COM—signal recorded at the output of the voltage comparator; (**b**) waveforms recorded at the output of the amplifier, peak detector, and voltage comparator, respectively.

The correctness of the peak detector and voltage comparator operation was tested in a measurement set-up shown in Figure 5a. As the AE signal source, the Olympus V101B ultrasonic probe was used, which was acoustically coupled to a piezoelectric transducer with a USG gel. The ultrasonic probe was excited by rectangular pulses with a voltage of 10 to 500 mV and a duration of 1 µs to 3 µs, which were generated using a Keysight DSOX2024A oscilloscope with a built-in waveform generator. The frequency with which the pulses were generated varied in the range from 1 Hz to 1 kHz. The outputs of the voltage follower, peak detector, and voltage comparator were connected with coaxial cables to channels 1, 2, and 3 of the oscilloscope, respectively. Examples of registered waveforms are shown in Figure 5b. In this case, the ultrasonic probe was excited by rectangular pulses generated with a frequency of 1 kHz, with a duration of 3 μs, and an amplitude of 50 mV.

#### 2.2.4. Single Board Microcontroller

The popular and inexpensive ($20) Teensy 3.2 development board featuring a 32-bit MK20DX256 microcontroller with the ARM Cortex-M4 core was used to count the PD pulses. The Teensy 3.2 module, despite its small dimensions (35 × 18 mm), has 34 digital input/output lines tolerant of 5V voltage, of which 12 can be used as PWM outputs, as well as one analog output. Between them, there are 21 high-resolution analog inputs (13 usable bits), of which 16 lines are shared with the digital ones and one with the analog output line (they cannot be used simultaneously). In addition, the board is equipped with 7 timers, 3 UART serial ports, SPI, I2C, I2S, CAN Bus, RTC module, 16 DMA channels, and touch sensor inputs. 

**Figure 6:**
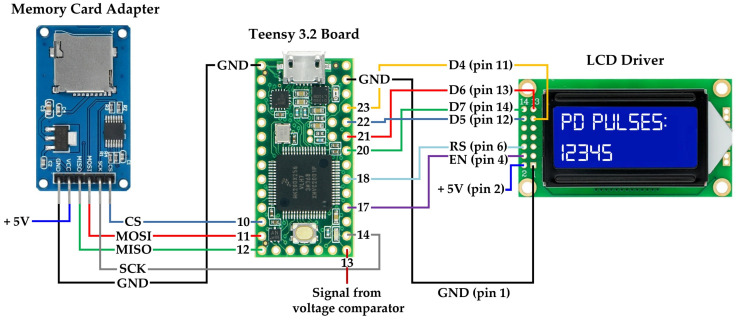
The schematic diagram of connecting the microSD card adapter and the 14-pin LCD driver to the Teensy 3.2 board.

The output signal from the voltage comparator is fed directly to Pin 13 of the microcontroller. If the amplitude of the signal at the input of the comparator is higher than the set trigger level, then its output returns a logic “high” state (~3.3 V). Otherwise, a logic “low” state (~0 V) is sent to Pin 13. The microcontroller continuously counts the PD pulses and records their current number every minute on the microSD memory card (Open Smart Technologies Limited, Hong Kong, China) and displays it on the 8 × 2 character monochrome LCD display type WC0802C (Hubbell Wiegmann, Freeburg, IL, USA). The schematic diagram of connecting the LCD driver and the microSD card adapter to the Teensy 3.2 board is shown in Figure 6.

#### 2.2.5. Power Supply

**Figure 7:**
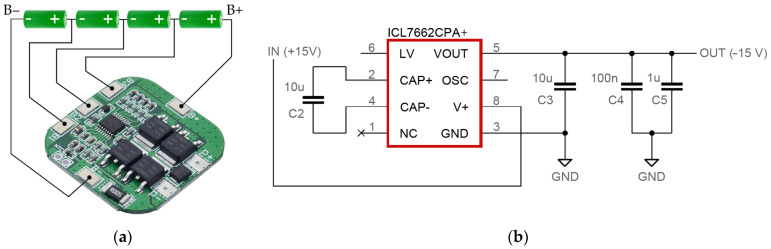
Components of the power supply system: (**a**) battery management system BMS 4S with the package of four series-connected lithium-ion cells US18650VTC5A; (**b**) electronic circuit diagram of the voltage converter ICL7662CPA+.

The monitoring system is powered by a package of four series-connected lithium-ion cells, type US18650VTC5A (Murata Manufacturing Co., Nagaokakyo, Japan), with a nominal voltage of 3.6 V and a capacity of 2600 mAh, which cooperate with a dedicated module of the BMS 4S battery management system (Figure 7a). The module has the function of charging cells with the option of a balancer, discharging, and a function protecting the cells against excessive discharge. The cells can be charged with a continuous current of up to 10 A. Since all digital modules (microcontroller, LCD driver, memory card adapter) require a voltage of 5 V, behind the battery management system, there is a step-down pulse converter module whose output voltage value is set with a potentiometer (from 1.0 V to 17 V). In turn, the instrumentation amplifier, voltage follower, and operational amplifiers used to build an active bandpass filter require a symmetrical voltage of 15 V. For this reason, the power module is additionally equipped with a voltage converter type ICL7662CPA+ (Figure 7b). This converter is a monolithic charge pump voltage inverter that converts a positive voltage in the range of +4.5 V to +20 V to a corresponding negative voltage of −4.5 V to −20 V. 

#### 2.2.6. Assembly of the System

**Figure 8:**
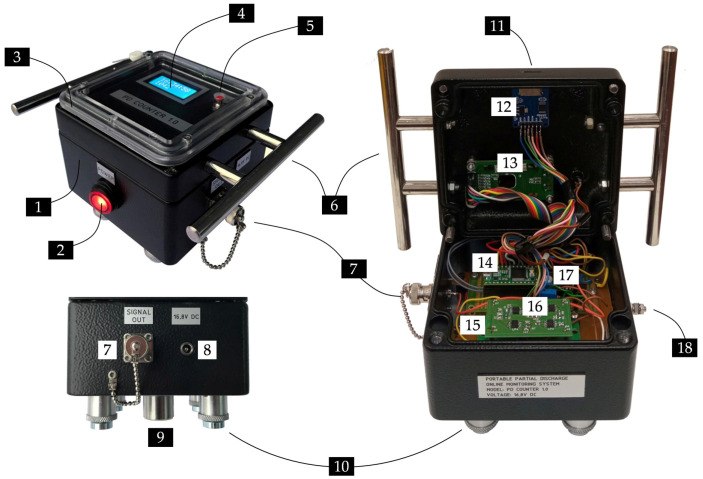
Photo of the system assembly: 1—aluminum electronic enclosure, 2—power switch, 3—polycarbonate inspection window, 4—LCD display, 5—reset switch, 6—handles, 7—BNC connector (bandpass filter output) for optional AE signal transmission, 8—DC power connector, 9—piezoelectric transducer, 10—magnetic holders, 11—micro SD card slot, 12—memory card adapter, 13—LCD driver board, 14—Teensy 3.2 development board, 15—amplifier board, 16—bandpass filter board, 17—peak detector and voltage comparator board, 18—grounding screw.

All electronic modules of the monitoring system together with the power supply system are placed in a waterproof (IP67) aluminum housing with dimensions of 120 × 120 × 75 mm. On the front panel of the system, there is an LCD screen and a switch for resetting the pulse counter. The front panel elements are protected by a transparent inspection window made of polycarbonate. In addition, the housing is equipped with a DC power connector through which the charger is connected, a micro SD card slot, a grounding screw, and a BNC connector for optional wired AE signal transmission (Figure 8).

### 2.3. Firmware

Each Teensy 3.2 module has a bootloader uploaded, thanks to which it can be programmed using the built-in USB connector (no external programmer required). Programs can be written in any environment supporting the C language or—as in the case of the discussed monitoring system—in the Arduino IDE programming environment with the Teensyduino extension installed. The computer program performs three main functions, i.e., counting PD pulses, displaying the number of pulses on the LCD display, and storing data (time, date, and number of pulses) on the memory card. The complete source code with comments can be found in the Appendix A.

## 3. System Testing

The test of the partial discharge monitoring system was carried out in three stages. In the first stage, the efficiency of the AE pulse counting function was checked using a signal generator and a piezoelectric transmitter. The second stage of the test was carried out in controlled laboratory conditions, during which partial discharges generated in the transformer tank model were monitored. The third stage of the test was carried out in field conditions, and the test object was a 10 MVA power transformer.

### 3.1. Testing the Efficiency of AE Pulse Counting

**Figure 9:**
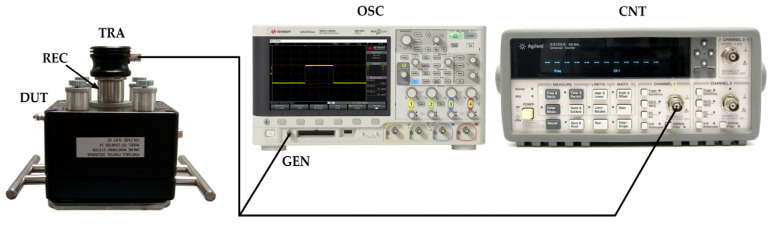
Schematic diagram of the measurement system for testing the efficiency of counting AE pulses by the monitoring system: TRA—wideband piezoelectric transducer Olympus V101B for generating AE pulses (transmitter), REC—piezoelectric transducer of the monitoring system (receiver), DUT—monitoring system (device under test), OSC—oscilloscope Keysight DSOX2024A, GEN—signal generator, CNT—universal counter Agilent/HP 53132A.

To assess the efficiency of counting AE pulses, a measurement system was used consisting of an oscilloscope Keysight with a built-in signal generator, a reference pulse counter, and a wideband piezoelectric transducer Olympus V101B, which was excited with rectangular pulses with a duration of 1 us and an amplitude of 100 mV (Figure 9).

The measurement results listed in Table 1 show that the system is characterized by high efficiency and is capable of lossless counting of up to 83,400 AE pulses per minute. Above this value, the counting accuracy decreases. 

### 3.2. Online Monitoring of Partial Discharges in Laboratory Conditions

**Figure 10:**
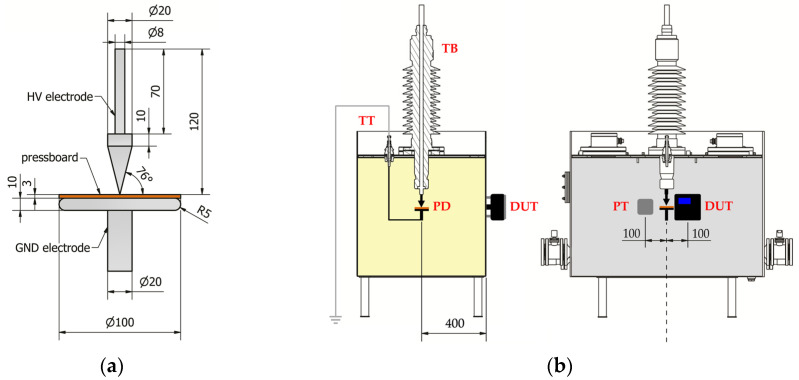
Test of the monitoring system in laboratory conditions: (**a**) electrode system for generating surface discharges; (**b**) arrangement of the tested and reference monitoring system on the transformer tank model: TT—oil filled transformer tank with electrode system for generating partial discharges; TB—transformer bushing; PD—electrode system for generating surface partial discharges PT—piezoelectric transducer A6890; DUT—system under test.

The tests were carried out in a shielded high-voltage laboratory using a model of a power transformer tank with dimensions of 1200 × 800 × 730 mm, which was filled with mineral oil. A system of electrodes for generating surface discharges on a round sample of pressboard with a diameter of 100 mm and a thickness of 3 mm was mounted to the internal connecting terminal of the bushing. The tip of a point electrode was located 400 mm from the wall of the ladle on which the tested system was installed. The PDtracker Portable system (Poznan University of Technology, Poznan, Poland) was used as a reference system, which is equipped with eight analog inputs with parallel sampling up to 20 MS/s. As partial discharge detectors, the system accepts both piezoelectric acoustic emission sensors (default configuration) and high-frequency current transformers. The AE sensor of the tested system and the A6890 sensor of the reference PDtracker system were placed exactly at the height of the PD source. The distance between the sensors was 200 mm, while the distance from the PD source to each sensor was the same and was about 400 mm (Figure 10). One of the basic functions performed by the PDtracker Portable system is also the counting of PD pulses. For this purpose, the system continuously records the signal, which is divided into time frames with a fixed width set by the user (in this case, 2 ms). If, in the analyzed time frame, the amplitude of the AE waveform exceeds the threshold value, then the system treats this event as the occurrence of a PD pulse and counts it. The threshold value is usually twice the average amplitude of the acoustic background noise, which in laboratory conditions does not exceed 10 mV. 

**Figure 11:**
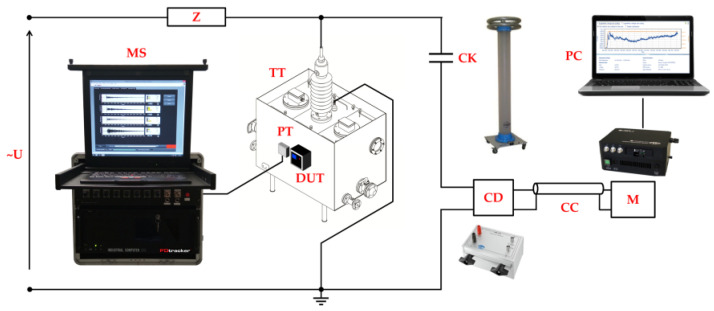
The measuring set-up used during testing of the monitoring system in laboratory conditions: U—high-voltage supply; Z—short-circuit current limiting resistor; MS—reference online PD monitoring system PDtracker; PT—piezoelectric transducer A6890; DUT—system under test; TT—oil filled transformer tank with electrode system for generating partial discharges; CK—coupling capacitor; CD—measuring impedance; CC—connecting cable; M—conventional partial discharge measuring device Doble PD-Smart; PC—computer.

During the tests, in addition to the reference PDtracker Portable system discussed above, a conventional PD meter (PD-Smart, Doble Engineering Company, Marlborough, MA, USA) and a measuring circuit compliant with the IEC60270 standard were also used (Figure 11). This made it possible to control the value of the apparent charge of the partial discharges and their intensity during the experiment. 

**Figure 12:**
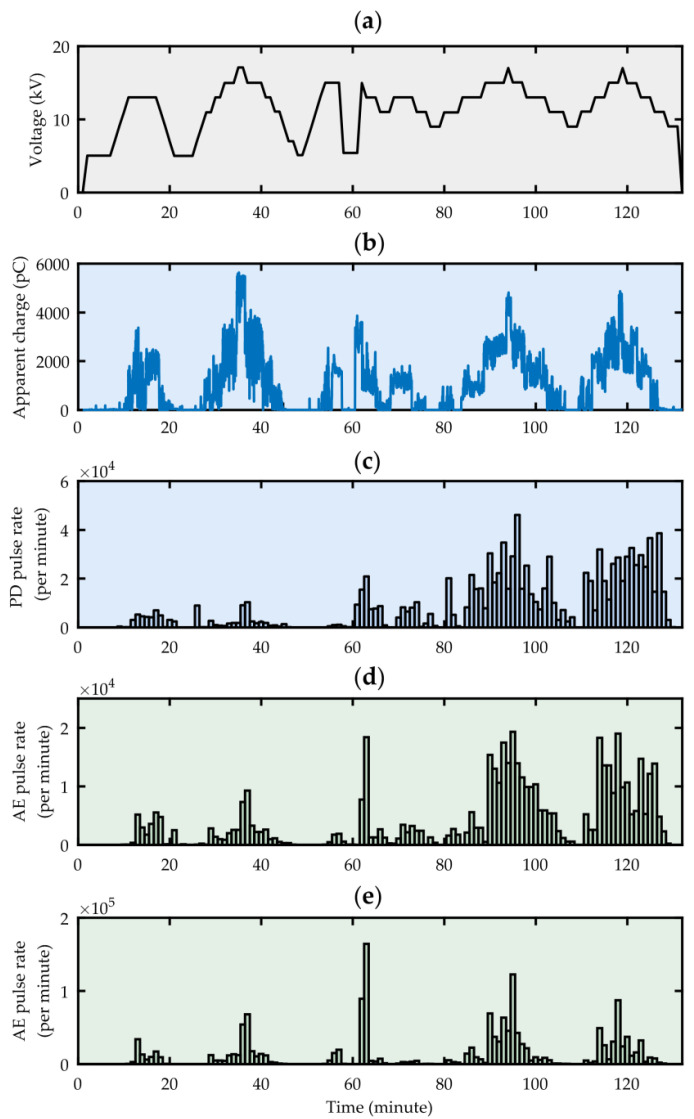
Test voltage value (**a**), PD apparent charge (**b**), number of PD pulses recorded by a conventional partial discharge meter PD-Smart, (**c**) number of AE pulses recorded by the reference monitoring system PDtracker Portable, (**d**) number of AE pulses registered by the tested, low-cost PD monitoring system (**e**).

A voltage in the range from 0 to 17 kV was applied to the electrode system, which allowed to generate partial discharge pulses of various intensity and energy. The inception voltage of surface discharges was *U_i_* = 9.2 kV, and their apparent charge ranged from 45 pC to over 5 nC. Exemplary test results are shown in Figure 12.

The analysis of the obtained test results shows that the discussed system is capable of online monitoring of partial discharges occurring in the oil–paper insulation system of the transformer. Due to the different implementations of the pulse counting procedure by the tested and reference acoustic emission systems, correlation analysis was performed instead of quantitative analysis. The Pearson correlation coefficient determined for the number of AE pulses recorded by both systems was 0.775 (strong correlation), while Spearman’s correlation coefficient was 0.91 (very strong correlation). In turn, the comparison with the number of PD pulses recorded by a conventional meter was more favorable for the PDtracker Portable reference system, as in this case, the Pearson correlation coefficient was 0.797, while for the low-cost monitoring system it was 0.639, which means a moderate correlation. Spearman’s correlation coefficient was 0.891 and 0.793, respectively, which can be interpreted as a strong correlation. 

### 3.3. Online Monitoring of Partial Discharges in a 10 MVA Power Transformer

**Figure 13:**
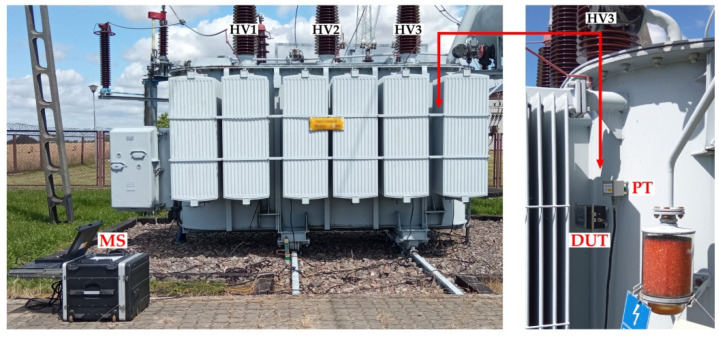
Monitoring of partial discharges in the HV3 phase of a 10 MVA power transformer: MS—reference monitoring system PDtracker Portable; PT—piezoelectric transducer of the reference monitoring system; DUT—tested low-cost PD monitoring system.

The field test of the system took place during partial discharge monitoring on a power transformer with a voltage of 115,000 ± 10% kV and a power of 10 MVA, which was manufactured in 1992. The reference measuring device was the eight-channel PDtracker Portable monitoring system, whose six piezoelectric transducers were placed near the phases of the low (LV1, LV2, LV3) and high (HV1, HV2, HV3) voltage sides. The last two transducers were installed on the side walls of the transformer tank (under the oil conservator and opposite the on-load tap-changer). The tested, low-cost PD monitoring system was always mounted in close proximity (approx. 15–20 cm) to the piezoelectric transducer of the reference system (Figure 13).

**Figure 14:**
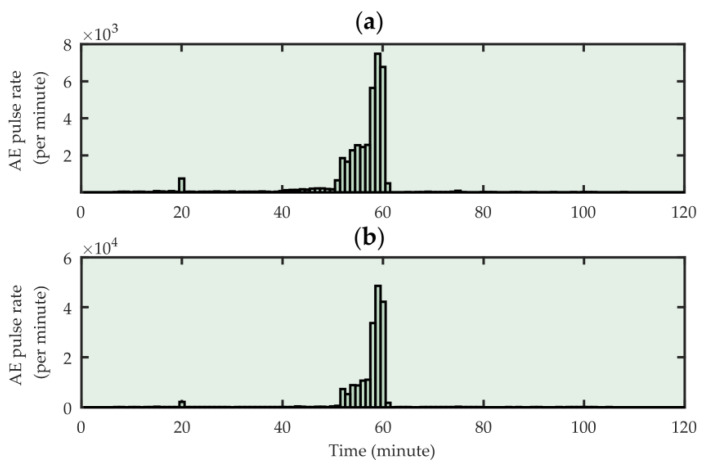
The number of AE pulses recorded during a 2-h partial discharge monitoring near the HV3 phase of a 10 MVA transformer by the reference PDtracker Portable system (**a**), and the tested low-cost PD monitoring system (**b**).

The measurement data analysis showed that in the tested transformer, both monitoring systems recorded a large number of AE pulses only near the HV3 phase (Figure 14). The period of increased intensity of the acoustic emission phenomenon lasted about 20 min and began at the moment when the OLTC (on-load tap changer) position changed. After another tap change, both systems recorded only single AE events. The Pearson correlation coefficient calculated for the number of AE pulses detected by both systems was 0.980 (very strong correlation), and the Spearman correlation coefficient was 0.869 (strong correlation). This showed that the performance of a low-cost PD monitoring system could be comparable to much more expensive and complex commercial systems.

## 4. Conclusions

The article presents in detail the design of a low-cost, portable online partial discharge monitoring system based on a non-invasive method of acoustic emission, which meets the guidelines of the IEC TS 62478 standard in terms of general requirements for the AE measurement system and for the measured PD quantities. The reason for developing this device was to eliminate the main obstacle to the widespread use of PD online monitoring systems, which is their high price and complicated operation, which requires a lot of experience. In the case of the discussed system, the total cost of all components used to build it does not exceed 300USD, which is a small fraction of the price of a commercial system. Thanks to this, the system can contribute to the dissemination of the idea of short-term PD monitoring, especially in developing countries, where with the dynamically growing demand for electricity, the demand for inexpensive, easy-to-manufacture transformer diagnostic systems is also growing. This system can also be used during laboratory measurements as a supplement or alternative to a stationary partial discharge detector. Another area of application for the device may be medium power transformers, for which online PD monitoring using complex measurement systems requiring additional infrastructure and qualified personnel is usually economically unjustified.

In addition to the low production price, the advantage of the system is:

- a high detection sensitivity of acoustic signals from partial discharges, resulting from the use of true differential AE sensor with optimized frequency response characteristics;
- the possibility of equipping the system with inexpensive modules dedicated to the Arduino/Teensyduino platform that increase its functionality, such as a Bluetooth module for wireless transmission of measurement data, or additional sensors to monitor other parameters of the transformer’s operation (e.g., an accelerometer for measuring transformer tank vibrations);
- easy installation on the transformer tank (thanks to the low weight and magnetic holders);
- no need to configure the device;
- high resistance to external electromagnetic interference due to the shielding of electronic modules and piezoelectric transducer elements.

The system also has some limitations, the most important of which is the battery power supply and the relatively short, several-day working time. Another disadvantage is related to the PD detection method used. In the case of the acoustic emission method, the sensitivity of partial discharge detection strongly depends on the distance between the AE sensor and the PD source. Therefore, before assembling the system, especially on large power transformers, it may be necessary to locate the place on the tank where the AE pulses are recorded in advance. A standard auscultatory technique (SAT) can be used for this purpose [48]. The possibility of real-time execution of only one task (counting PD pulses) can also be considered a disadvantage of the system. This is due to the limited computing power of the Teensy 3.2 microcontroller, which ranges from a few to several dozen MIPS. However, this problem can be relatively easily solved by equipping the system with a second microcontroller or using—unfortunately at the cost of many times more power consumption—a microcontroller with a much more efficient processor, such as Teensy 4.1 with an ARM Cortex-M7 processor clocked at 600 MHz.

## Figures and Tables

**Table 1:** The results of the AE pulse counting performance test by the monitoring system.

Pulse Generation Frequency (Hz)	The Number of Pulses Generated per Minute	Average Amount of Lost Pulses (%)	Maximum Amount of Lost Pulses (%)
≤1380	≤83,400	0	0
1400	84,000	0.60	0.79
1500	90,000	2.99	4.08
1600	96,000	4.74	6.23
1700	102,000	7.91	8.28

## Data Availability

Not applicable.

## Appendix A. Source Code for the Firmware of the Online PD Monitoring System

//declare libraries

#include <TimeLib.h> //RTC library

#include <FreqCount.h> //reading pulses library

#include <LiquidCrystal.h> //LCD library

#include <SPI.h> //Serial Peripheral Interface library

#include <SD.h> //SD card

//declare LCD pinout

const int rs **=** 18, en **=** 17, d4 **=** 23, d5 **=** 22, d6 **=** 21, d7 **=** 20;

LiquidCrystal lcd**(**rs, en, d4, d5, d6, d7**)**;

//declare count variable

unsigned long count **=** 0;

//declare count in previous iteration variable

unsigned long prior_count**=**0;

//auxiliary variable

bool x **=** 0;

File myFile;

//define pin connected to "RESET" button

const byte resetPin **=** 15;

//void setup is executing only once, after start

void setup**()**  **{**

  //PULLDOWN the NC "RESET" button

  pinMode**(**resetPin, INPUT_PULLDOWN**)**;

  //set SCK in SPI connection

  SPI.setSCK**(**14**)**;

  SPI.begin**()**;  

  setSyncProvider**(**getTeensy3Time**)**;

  //begin frequency counting 

  FreqCount.begin**(**100**)**;

  //define LCD size 

  lcd.begin**(**8, 2**)**;

  Serial.begin**(**115200**)**;

  //try to connect to external RTC and sync

  **if** **(**timeStatus**()!=** timeSet**)** **{**

    Serial.println**(**"Unable to sync with the RTC"**);**

  **}** **else** **{**

    Serial.println**(**"RTC has set the system time"**)**;

  **}**

//setup message  

Serial.print**(**"Initializing SD card..."**);**

lcd.setCursor**(**0, 0**);**

lcd.print**(**"SD:"**);**

//connect to SD, if failed print a message

**

**if**** **(!**SD.begin**(**10**))** **{**

Serial.println**(**"Initialization failed!"**)**;

lcd.setCursor**(**4,0**);**

lcd.print**(**"FAIL"**)**;

**

**}**

**

**

**else**** ****{**

**

Serial.println**(**"Initialization done."**);**

lcd.setCursor**(**4,0**);**

lcd.print**(**"OK"**);**

**

**}**

**

//starting delay after initialization to make a communicate readable

delay**(**4000**);**

**

**}**

**

//void loop is running repeatedly

void loop**()** **{**

  **if** **(**FreqCount.available**())** **{**

    count **=** count **+** FreqCount.read**();**

    //if the number of counted pulses changed print a new one on the LCD

    **if** **(**count **!=** prior_count**)** **{**

      //next line can be uncommented if the console connection is enabled

      //Serial.println(count);

      //print the number of counted pulses

      lcd.setCursor**(**0, 1**);**

      lcd.print**(**count**);**

      //overwrite the number of pulses

      prior_count **=** count;

    **}**

  **}**

  //print the time on the LCD

  lcd.setCursor**(**0, 0**);**

  printDigitsLcd**(**hour**());**

  lcd.print**(**":"**);**

  printDigitsLcd**(**minute**());**

  lcd.print**(**":"**);**

  printDigitsLcd**(**second**());**

  //save number of pulses to file in 1 minute intervals (seconds==0)

  //bool x is to avoid saving more than once in one second

  **if** **(**second**()==**0**&&**x**==**0**){**

  digitalClockDisplay**()**;

  x**=**1;

  //declare file name and open it

  myFile **=** SD.open**(**"count.txt", FILE_WRITE**);**

//save time and number of pulses to the file

**

**if**** **(**myFile**)** **{**

Serial.print**(**"Writing to count.txt... "**);**

myFile.print**(**day**());**

  myFile.print**(**"."**);**

  myFile.print**(**month**());**

  myFile.print**(**"."**);**

  myFile.print**(**year**())**;

  myFile.print**(**" "**);** 

  myFile.print**(**hour**())**;

  printDigitsFile**(**minute**())**;

  printDigitsFile**(**second**())**;

  myFile.print**(**","**)**;

  myFile.print**(**count**)**;

  myFile.println**();** 

**
**}**
**

myFile.close**();**

**
**}**
**

  //if the second turn to 1, overwrite x value as 0

  **if** **(**second**()==**1**){**

  x**=**0;

  **}**

//execute function after the button "RESET' is pushed

**

**if**** ****(**** digitalRead**(**resetPin**)==**0**)**

** **{****

  zero**()**;

** **}****

**
**}**
**

//save data in a text file; "PULSES:" and number of pulses counted

void digitalClockDisplay**()** **{**

  Serial.print**(**day**())**;

  Serial.print**(**"."**)**;

  Serial.print**(**month**())**;

  Serial.print**(**"."**);**

  Serial.print**(**year**())**;

  Serial.print**(**" "**)**; 

  Serial.print**(**hour**())**;

  printDigits**(**minute**())**;

  printDigits**(**second**())**;

  Serial.print**(**" "**);**

  Serial.print**(**"PULSES: "**)**;

  Serial.print**(**count**)**;

  Serial.println**()**; 

**
**}**
**

//get time from RTC

time_t getTeensy3Time**()**

**
**{**
**

  **return** Teensy3Clock.get**()**;

**}**

#define TIME_HEADER “T”   

unsigned long processSyncMessage**()** **{**

  unsigned long pctime **=** 0L;

  const unsigned long DEFAULT_TIME **=** 1357041600; // Jan 1 2013 

  **if****(**Serial.find**(**TIME_HEADER**))** **{**

     pctime **=** Serial.parseInt**()**;

     **return** pctime;

     **if****(** pctime **<** DEFAULT_TIME**)** **{** //check if the time if valid

       pctime **=** 0L;     //return 0 if the time is invalid

     **}**

  **}**

  **return** pctime;

**
**}**
**

//add 0 when digits<10 (Serial output)

void printDigits**(**int digits**){**

  Serial.print**(**":"**)**;

  **if****(**digits **<** 10**)**

    Serial.print**(**'0'**)**;

  Serial.print**(**digits**)**;

**
**}**
**

//add 0 when digits<10 (LCD output)

void printDigitsLcd**(**int digits**){**

  **if**(digits **<** 10**)**

    lcd.print**(**'0'**)**;

  lcd.print**(**digits**)**;

**
**}**
**

//add 0 when digits<10 (file output)

void printDigitsFile**(**int digits**){**

  myFile.print**(**":"**)**;

  **if****(**digits **<** 10**)**

    myFile.print**(**'0'**)**;

  myFile.print**(**digits**)**;

**
**}**
**

//function for resetting the number of pulses

void zero**()** **{**

  count**=**0;

  lcd.setCursor**(**0, 1**)**;

  lcd.print**(**"        "**)**;

  delay**(**500**)**;

**
**}**
**
